# Population-specific long-range linkage disequilibrium in the human genome and its influence on identifying common disease variants

**DOI:** 10.1038/s41598-019-47832-y

**Published:** 2019-08-06

**Authors:** Leeyoung Park

**Affiliations:** Natural Science Research Institute, Yonsei University, Seoul, 120-749 Korea

**Keywords:** Genetic variation, Genetic variation

## Abstract

Despite the availability of large-scale sequencing data, long-range linkage disequilibrium (LRLD) has not been extensively studied. The theoretical aspects of LRLD estimates were studied to determine the best estimation method for the sequencing data of three different populations of African (AFR), European (EUR), and East-Asian (EAS) descent from the 1000 Genomes Project. Genome-wide LRLDs excluding centromeric regions revealed clear population specificity, presenting substantially more population-specific LRLDs than coincident LRLDs. Clear relationships between the functionalities of the regions in LRLDs denoted long-range interactions in the genome. The proportions of gene regions were increased in LRLD variants, and the coding sequence (CDS)-CDS LRLDs showed obvious functional similarities between genes in LRLDs. Application to theoretical case-control associations confirmed that the LRLDs in genome-wide association studies (GWASs) could contribute to false signals, although the impacts might not be severe in most cases. LRLDs with variants with functional similarity exist in the human genome indicating possible gene-gene interactions, and they differ depending on populations. Based on the current study, LRLDs should be examined in GWASs to identify true signals. More importantly, population specificity in LRLDs should be examined in relevant studies.

## Introduction

Long-range linkage disequilibrium (LRLD), which is the linkage disequilibrium (LD) between distant variants within a chromosome, can occur due to genetic drift or gene interactions in a chromosome or population substructure^1,2^. Demographic changes can influence LD, and reduced LD is observed during rapid population growth^3^. In contrast, recurrent severe bottlenecks can extend LD between distant genomic regions^4^. However, such impacts would appear as long chromosomal regions harboring pairs of both close and distant variants, and these effects will always be more substantial in close regions than in distant regions, as shown in a previous study on a bottleneck of the European population using LD over a 2-Mb region in chromosome 17^5^. LRLD can also be induced due to random sampling of the genomes in a sampled population. The haplotype frequency of two variants is limited to the allele frequencies of the two variants^6^. When the minor allele frequencies of one variant are very small relative to the sample size, the haplotype frequency at equilibrium is biased at one side and generates LD by chance. Therefore, unless the sample size is sufficiently large or the minor allele frequencies of the two variants are sufficiently high relative to the sample size, LD between two variants can be observed even at long distances by chance.

A recent study of the human genome indicated that LRLD occurs at a higher rate than expected^7^, which introduced a new statistic, *p*_*D*_^*max*^, based on Fisher’s exact test to measure whether two LD blocks in a chromosome contain variants that exhibit LRLD between the blocks. If the sample size is small, one side of the limited haplotype frequencies results in high LD when the haplotype frequency at equilibrium is close to one of the edges^6^, which was the case in the previous study using 60 individuals from the Yoruba in Ibadan, Nigeria (YRI), obtained from HapMap data^7,8^. Therefore, tests using sequencing data of larger sample sizes and excluding rare variants are required to examine the true patterns of LRLD. The whole-genome sequencing data of the 1000 Genomes Project have been selected for examining LRLD patterns in the human genome^9,10^. Previous works have demonstrated population differences in allele frequencies and LD, which can also induce differential LRLD between populations. Nonetheless, no efforts have been made to examine population differences in LRLD patterns. LRLDs that are coincident in all populations would indicate the benefits of the occurrence of LRLD induced by population-wide gene interactions.

Subpopulation structure can produce spurious LRLD^2,11^, which is observed in human populations^12^. No significant impact of population substructure was expected within a continent, considering the small mean allele frequency differences between 0.0044 and 0.016 among different European populations^13^, although there is an argument in African populations^14^. In addition, based on previously introduced equations^2^, LRLD due to subpopulation structure might not be serious as long as the differences in allele frequencies are not substantially large, which was examined in the current study. LRLD also has the potential to identify functional variants, provided the LRLD is not a confounded result due to subpopulation structure or bottlenecks. Gene interactions cause LD^1^, and the existence of LRLD between functional variants can provide evidence of long-range gene interactions. Notably, this type of gene interaction testing does not involve any phenotype, although associated phenotypes should eventually be identified. Previous studies have utilized numerous approaches to study gene interactions associated with diseases^15^. Exhaustive approaches for basic allelic and/or genotypic association may be applicable to detect gene interactions without disease status^16,17^. As an allelic association, LD is a good basic indicator of gene interactions. LRLD might also influence the results of genome-wide association studies (GWASs)^18^. Based on a traditional yet robust method, the current study identified the actual LRLD in three different human populations of the 1000 Genomes Project, and the possible impacts of LRLD on GWASs were examined.

## Results

### Theoretical properties of testing long-range linkage disequilibrium

Both Fisher’s exact test, which has been previously used to detect LRLD^17,19^, and the chi-square statistic (see Eq. 1) can be used to test the hypothesis of no disequilibrium^17^.

1 $$\begin{array}{rcl}{\hat{D}}_{AB} & = & {\tilde{p}}_{AB}-{\tilde{p}}_{A}{\tilde{p}}_{B}\\ {X}_{AB}^{2} & = & {z}^{2}=\frac{2n{\hat{D}}_{AB}^{2}}{{\tilde{p}}_{A}(1-{\tilde{p}}_{A}){\tilde{p}}_{B}(1-{\tilde{p}}_{B})}\end{array}$$

when AB is a haplotype consisting of two major alleles, the frequency, pAB, must be between 1-pa-pb and 1-max(pa, pb), where pa and pb indicate the minor allele frequency of each variants. pAB at the state of linkage equilibrium is not symmetrically positioned between the possible minimum and possible maximum^6^. The severity of asymmetry is largest when the allele frequencies approach 0 or 1. If the sampling size is sufficiently large relative to the allele frequencies, this restriction of pAB is not a significant concern. However, when the sample size is small and/or the equilibrium value of pAB is severely skewed to one of the boundaries, random sampling with small sample sizes results in strong LD by chance. Therefore, to avoid this situation, sufficiently large samples sizes and variants with appropriate allele frequencies must be considered.

**Figure 1:**
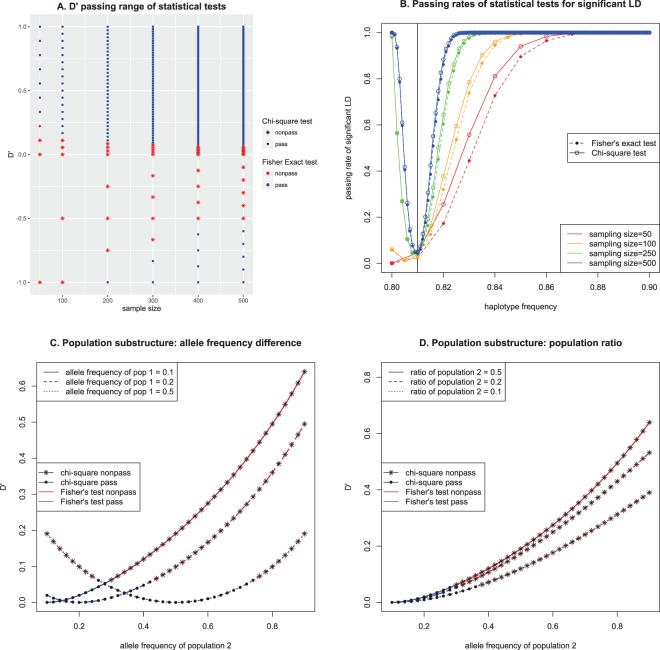
Theoretical comparisons of LD between Fisher’s exact test and the chi-squared test (pass: significant p-values) when allele frequencies of both variants are 0.1: (**A**). Statistical test results depending on D′ and sample sizes depending on available haplotype frequencies; (**B**). Simulation results for various sample sizes for 10,000 times; (**C**). Impact of population substructure depending on the allele frequencies of equal-sized populations (both loci in a population have the same allele frequency); (**D**). Impact of population substructure depending on the ratios of the second population when the allele frequency of first region is 0.1 for both populations.

These issues are relevant to both Fisher’s exact test and the chi-square test. Figure 1 indicates the test results (one-tailed, level α = 0.05) when both allele frequencies are 0.1 for various sample sizes. Haplotype frequencies with intervals of 1/(2 N) between 0.8 and 0.9 were examined for various sample sizes (N) in Fig. 1A to determine whether a significant p-value is identified based on the linkage equilibrium test. As shown in Fig. 1A, when the sample size is small, the tests cannot detect LD over a broad range even when D′ equals 1^20,21^. Fisher’s exact test reveals a larger range of linkage equilibrium detection for all sample sizes. Based on random sampling of fixed haplotype frequencies using in-house scripts with basic functions in the R statistical package, the simulations were conducted 10,000 times to determine the percentage of detection of LD for a given haplotype consisting of two major alleles that can be directly converted to D′. As shown in Fig. 1B, LD is not accurately detected in either test when the sample size is small. This result suggests that the small sample size of the previous study (60 individuals with 120 haplotypes)^7^ likely led to biased results. Similar to the findings presented in Fig. 1A, the spurious detection of LD due to small sample sizes and extreme allele frequencies was consistently slightly more severe for Fisher’s exact test, whereas the chi-square test consistently exhibited slightly less spurious detection of LD.

To examine the influence of population substructure, the LD of the total population was studied for various subpopulation structures at linkage equilibrium. Similar to Fig. 1A, C presents the theoretical results of both the chi-square and Fisher’s exact tests with a fixed allele frequency of the first population and various allele frequencies of the second population for a total of 500 individuals, which combined two different equal-sized populations. The results indicated that the impact of subpopulation structure might not be sufficiently large to be considered LD using those tests unless the differences in allele frequencies for the two variants between the populations are at least greater than 0.2, even for the best scenario for a spurious LD. As shown in Fig. 1D, the impact also diminished as the ratio of the second population decreased. Therefore, a combined population of very similar populations results in small differences in allele frequencies that do not generate significant LRLD artifacts due to the population substructure.

### Long-range linkage disequilibrium in the human genome

**Figure 2:**
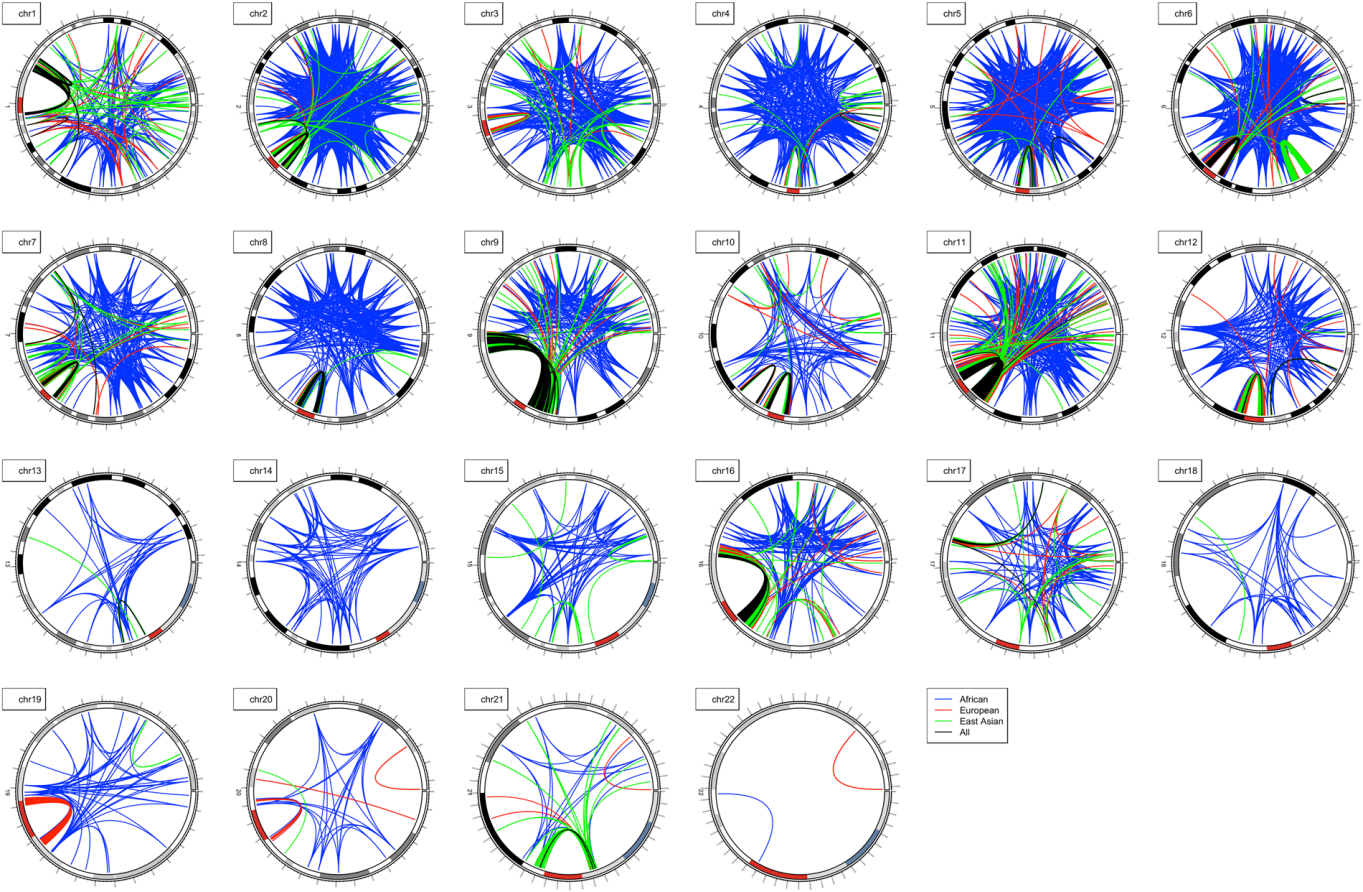
Chromosome-wide LRLD of each population of AFR, EUR, and EAS and coincident LRLD in all three populations (the red bar indicates the centromere).

Each population exhibited mostly distinctive, population-specific LRLD (Fig. 2), although common LRLD also existed in a number of regions. Most of the common LRLD among the three populations was observed surrounding the centromeric region. The results are consistent with the reduced meiotic recombination on and near the centromere^22^ and indicate that the calculations performed at the genome-wide level are accurate. With the exception of chromosomes 13, 14, 15, 17, 18, and 22, dense LRLD was observed surrounding the centromere. Chromosomes 3, 4, 19, and 20 showed LRLD surrounding the centromere that was specific to certain populations, i.e., not common among all three populations. These phenomena can be observed in greater detail in Supplementary Fig. 1 and Table 1. As shown in Table 1, the number of detected LRLDs was considerably reduced when excluding LRLDs with positions either on or near the centromere (±5,000,000 bp).

**Table 1:** Number of LRLDs by population (expected: if independent, the expected number of intersections).

	Total							Without centromeric region ± diff						
	AFR	EUR	EAS	AFR& EUR	AFR& EAS	EUR& EAS	ALL	AFR	EUR	EAS	AFR&EUR	AFR&EAS	EUR&EAS	ALL
1	15777	49316	60996	3963	3827	22411	2948	1243	9767	25922	715	521	5215	415
2	13498	66239	20710	5655	2419	12344	1867	747	789	904	165	172	593	165
3	1877	57659	952	2	0	18	0	317	40	711	2	0	18	0
4	2570	235	4246	24	46	34	18	450	218	663	24	46	34	18
5	19883	74065	6481	1746	450	835	93	770	849	3649	94	225	510	87
6	658700	1323757	869597	275263	196244	533885	146681	397	33811	22257	41	40	171	38
7	73047	170435	88816	20610	8928	27003	3910	253	246	152	7	5	64	5
8	19327	13385	7360	5658	3113	4173	2411	303	0	10	0	0	0	0
9	568926	531978	715387	366786	375228	411798	309129	2062	5930	14860	556	832	3298	508
10	63940	188802	120079	16887	9672	48558	5474	176	115	241	4	11	6	0
11	5666979	7974596	8412600	3640925	3970719	5902528	3063194	226	13	339	0	0	0	0
12	67713	236888	1320	22680	440	299	136	163	14	5	0	0	0	0
13	224	2213	2199	190	190	1824	190	19	0	0	0	0	0	0
14	75	0	0	0	0	0	0	71	0	0	0	0	0	0
15	100	133	247	2	4	41	0	99	4	176	2	3	2	0
16	2758	39352	19734	1065	1022	5683	729	159	480	169	0	0	158	0
17	2540	42	2550	23	2002	21	21	2507	39	2536	23	2002	21	21
18	40	0	3	0	0	0	0	11	0	0	0	0	0	0
19	1986	31992	30	0	0	0	0	6	0	30	0	0	0	0
20	42	109	1	2	0	0	0	17	12	0	0	0	0	0
21	2116	3868	4532	110	136	268	9	6	1	0	0	0	0	0
22	1	12	0	0	0	0	0	0	12	0	0	0	0	0
Sum	7182119	10765076	10337840	4361591	4574440	6971723	3536810	10002	52340	72624	1633	3857	10090	1257
Expected				2733462	2624978	3934506	999047				3079	5382	28164	2087

Upon first inspection of Fig. 2, the highest number of LRLD detections appeared in AFR; however, as shown in Table 1, the actual number of LRLD detections is substantially increased in EUR and EAS compared with AFR. This discordance is due to the existence of often dense LRLD regions in EUR and EAS, possibly due to past population history as further addressed in the Discussion. Each chromosome showed a different number of LRLDs, which was uncorrelated with chromosome size, as shown in Table 1. High numbers of LRLDs were identified at positions surrounding the centromeres, as shown in Fig. 2, especially in chromosomes 6 and 11. Even after excluding the centromeric regions, EUR and EAS showed large numbers of LRLD detections in chromosomes 1, 6, and 9. In particular, chromosome 9 showed a large number of LRLDs specific to EAS. Notably, some of the chromosomes have little or no LRLD depending on the population, even when considering size. The severely uneven numbers of chromosomal LRLDs confirm that genome-wide confounding due to population substructures or bottlenecks might not be the main source of LRLDs observed in the current study.

Based on the African Genome Variation Project^23^, the mean pairwise F_ST_ among sub-Saharan African populations was 0.019, which made it difficult to explain the diverse LRLD patterns of AFR presented in Fig. 2. They observed many variants that were subpopulation specific. As the current study utilized the common variants in all three populations, the number of subpopulation-specific variants might be minimal. To examine the impact of subpopulation-specific variants on the LRLD patterns in AFR, the allele frequencies of subpopulations of AFR, EUR and EAS were examined. The mean differences in subpopulation allele frequencies were similar, i.e., 0.058 in AFR subpopulations, 0.051 in EUR subpopulations and 0.059 in EAS subpopulations. Among the AFR LRLD variants, 814 (2.15%) showed an allele frequency difference greater than 0.5 between two subpopulations. The allele frequencies of those extreme variants showed a differentiation of two groups, ESN/GWD/MSL and YRI/LWK, with possible influences on LRLDs. However, the proportion of LRLDs harboring at least one of the 814 variants was 0.00158, indicating that the impact of these variants on the diverse LRLD pattern in AFR is limited. The allele frequencies of those subpopulation-specific LRLD variants were much more similar between ASW/ACB and YRI/LWK than between ASW/ACB and ESN/GWD/MSL, possibly supporting previous results of recent population changes and slave trades^24^.

As shown in Table 1, the numbers of positions exhibiting common LRLD between two populations or among all three populations are larger than the expected number if LRLD detections are independent in each population. However, as shown in Supplementary Fig. 1, common LRLDs are primarily located in LRLD positions surrounding the centromere, indicating that there are more genome-wide population-specific LRLD positions. In addition, when removing the regions on and near the centromere, the number of common LRLDs between two or three populations is fewer than expected, suggestive of population-specific, long-range interactions. Interestingly, AFR showed LRLD in more varied positions than EUR and EAS. This observation potentially reflects the different population histories of AFR from EUR and EAS, which might maintain a higher diversity of weak interactions between regions at long distances.

### LRLD structural variants in the human genome

Structural variants, especially large inversions, can influence LRLD. As shown previously^10^, the majority of structural variants in the data of the 1000 Genomes Project are very rare, resulting in a small proportion of structural variants in the current analyses, most of which are copy number variations (CNVs) with lengths larger than 1 Mb^25^. In the current study, given that the minimum distance between variants in LRLD is 5 × 10^6^ bp, common long CNVs might be responsible for the detected LRLDs. The number of LRLDs that include at least one structural variant was 47,703 among the 15,914,091 LRLDs detected in at least one population. Interestingly, only 74 structural variants (55 CNVs and one inversion) were responsible for all the LRLDs with at least one structural variant, as summarized in Supplementary Dataset 1. However, the longest length among the structural variants was 59,232 bp, which is substantially smaller than the minimum LRLD distance of 5 × 10^6^ bp, in the current study. This result indicates that most of these structural variants are LRLD hotspots (Supplementary Dataset 1A) that might not influence LRLD considering their lengths.

The structural variants of LRLD were mostly located in nongenic regions (42) and noncoding gene regions (31). One structural variant (CNV) of LRLD in EAS (allele frequency 0.26) was located in the intron region of *ZNF705G* (zinc finger protein 705G) on chromosome 8. Given that the length of the structural variant was 14,839, which is much larger than the gene length (4,992 bp), the variant likely affects gene function. The variant was in LRLD with four variants of the pseudogene, *ENPP7P6* (ectonucleotide pyrophosphatase/phosphodiesterase 7 pseudogene 6) with allele frequencies of 0.73, 0.17, 0.16, and 0.22 in EAS. As shown in Supplementary Dataset 1B, the LRLDs with structural variants again showed substantial population specificity and varied depending on the chromosomes. Although the number of concurrent LRLDs was substantially larger than the expected number, the numbers of concurrent LRLDs between two populations was slightly less than the expected values. The number of LRLDs with both structural variants was 30, all of which were surrounding the centromere in chromosome 11.

To examine the direct impacts of structural variants, the overlaps between LRLD variants and structural variants were studied. For a total of 55,669 LRLD variants, 24,572 LRLD variants (44.1%) were located within structural deletion or inversion variants. There are severe chromosomal variations in their percentages from 6.5% of chromosome 15 to 71% of chromosome 13. On the contrary, only 19 LRLD variants (0.034%) were located on insertion structural variants. For all variants that were not structural variants of the 1000 Genomes Project, 19,685,604 variants (24%) were located within deletion or inversion variants among 81,119,003 variants, and 604 variants (0.00074%) were located on insertion structural variants. The differences in percentages indicated that LRLD variants were more likely located on structural variants.

The actual number of structural variants harboring 24,572 LRLD variants was 1,039, with a mean value of length 48,324 bp from the minimum of 310 bp to the maximum of 1,747,249 bp. The combined lengths of structural variants (50,208,184 bp) may cover the substantial proportion of the genome, inevitably making that many variants are located on the structural variants. As expected, their allele frequencies were very small with mean allele frequencies of 0.0234, 0.0289, and 0.0284 for AFR, EUR and EAS, respectively. Among 1,039 structural variants, only 75, 84, and 87 (7.2%, 8.1%, and 8.4%) for AFR, EUR, and EAS respectively were minor allele frequencies larger than 0.1. The mean lengths of those common structural variants with minor allele frequencies higher than 0.1 was 20,966 bp (the maximum of 255,314 bp on chromosome 16), which were difficult to cover the chromosome-wide LRLDs. It is likely that some structural variants influenced the LRLD formations, yet probably not most of them.

### Gene properties of LRLD in the human genome

**Figure 3:**
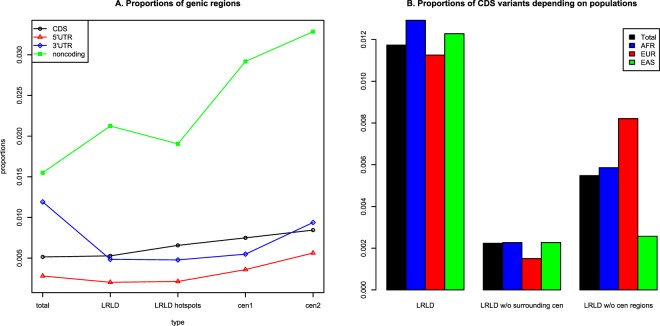
Proportions of variant types: (**A**). Proportions of variants in CDS, 5′UTR, 3′UTR, and noncoding gene regions among the total variants, LRLD variants in at least one population, LRLD variants excluding LRLDs surrounding the centromere (cen1), and LRLD variants with at least one region on or near the centromere (cen2); (**B**). Proportions of CDS variants depending on populations for LRLD variants, cen1 and cen2.

The number of calculated variants for LRLD is summarized in Supplementary Table 1, and Fig. 3 presents the proportions of each genic variant. In Fig. 3A, the proportion of coding sequence (CDS) variants was 0.00515 for the total calculated variants and increased in the following order: LRLD variants (0.00528), LRLD hotspots (0.00657), LRLD variants excluding those surrounding the centromere (0.00749), and LRLD variants excluding those on and near the centromere (0.00845). Noncoding genic regions showed a more dramatic increase in LRLDs. The lower proportion of LRLD hotspots than that of total LRLDs may indicate specific gene regulatory interactions rather than global regulations. Although the effects were not clear as those of CDS, the 5′UTR and 3′UTR also exhibited trends similar to CDS among LRLD groups. The proportion of CDS functional variants including any frameshift, missense, inframe deletion, stop codon, and splice variants showed a clear increase from 0.476 of the total calculated CDS variants to 0.650. Interestingly, 91% of the functional variants were observed more than once in LRLDs, indicating the presence of LRLD hotspots in at least a number of the cases. The exact number of proportions and detailed descriptions involving nonsense-mediated decay (NMD) transcript variants are presented in Supplementary Table 2 and Supplementary Text, which provides evidence that there are many more LRLDs between a genic region and a nongenic region for LRLDs excluding those surrounding centromeres and functional LRLD variants are likely hotspots. The proportions of CDS also differed depending on populations, especially for LRLDs excluding centromeric regions (Fig. 3B), indicating that the possible functional relevance may differ depending on the populations.

The total number of CDS-CDS LRLDs was 1,518, and 98% of these LRLDs (1490) were on chromosome 11 (Supplementary Dataset 2), closely surrounding the centromere (Fig. 2). This result is reasonable given that the vast majority of centromeric LRLDs were on chromosome 11 as shown in Table 1, and the excessive number of LRLDs surrounding the centromere might represent gene interactions before and after the centromere. Among the 1490 LRLDs of CDS-CDS, 780 were functional. Interestingly, the entire region before and after the centromere harbors olfactory receptor family genes^26^ that are in LRLDs through functional variants mostly involving missense, stop, and frameshift variants, as shown in Supplementary Dataset 2. The remainder of CDS-CDS LRLDs that were both functional were on chromosomes 1 (1), 2 (9), 5 (1), 6 (2), 9 (4), and 16 (2) (the number of LRLDs is indicated in parentheses).

Among the CDS-CDS interactions, the LRLDs that occurred in all three populations were on chromosome 9 (3) and 11 (159). The LRLDs on chromosome 9 were missense-missense interactions. In this case, three missense variants on spermatogenesis-associated 31 subfamily A member 6 (*SPATA31A6*) were in LRLDs with a missense variant on ankyrin repeat domain 20 family member A4 (*ANKRD20A4*), with allele frequencies of 0.40, 0.30, and 0.32, respectively for AFR, EUR, and EAS. The former is expressed exclusively in the testis, and the latter is expressed mostly in the testis^27^. As shown in Supplementary Fig. 2, the LD between common variants (minor allele frequency greater than 0.1) in the region ±5,000 bp of the LRLD variants showed that the LRLD variants were in LD blocks representing strong LRLD between the variants and nearby variants. Gene enrichment analysis of the LRLD variants in CDSs revealed two biological processes: detection of chemical stimulus involved in sensory perception of smell (false discovery rate (FDR) p-value of 6.38 × 10^−23^) and G-protein-coupled receptor signaling pathways (FDR p-value of 1.76 × 10^−10^)^28,29^.

The remainder of the functional CDS-CDS LRLDs were population specific. For chromosome 1, the missense-missense LRLD between DnaJ heat shock protein family member C8 (*DNAJC8*) and WD repeat domain 3 (*WDR3*), which are both involved in cell cycle progression^30,31^, was exclusively observed in AFR, based on FDR-corrected p-values. Without FDR corrections, the p-values for EUR and EAS were 0.066 and 0.074, respectively. For chromosome 2, LRLDs surrounding the centromere were observed in EUR and/or EAS, and the LRLDs that were significant in only one population showed LRLD trends in the alternative population. For chromosome 6, the functional CDS-CDS LRLDs were located in the coincident dense LRLD region of both EUR and EAS; however, those functional LRLDs were significant only in EUR after FDR corrections. For the two functional CDS-CDS LRLDs, one variant was an NMD transcript variant, and the other two interacting variants were missense variants as predicted by Perl Application Program Interface (API) ENSEMBL^32^. However, one of the missense variants was synonymous based on evaluation on the National Center for Biotechnology Information (NCBI) website. The entire region is a major histocompatibility complex (MHC) class II region, indicating possible gene interactions related to the immune system specifically in EUR. Finally, in chromosome 16, the functional CDS-CDS LRLDs included an NMD transcript variant in essential meiotic structure-specific endonuclease subunit 2 (*EME2*) and two variants (one synonymous harboring frameshift variant at the same position/ one missense) in splicing factor 3b subunit 3 (*SF3B3*) in EUR (and AFR without FDR correction). Interestingly, both genes are involved in DNA repair, as indicated in the NCBI gene summary.

### Impact of LRLD on case-control association studies

**Figure 4:**
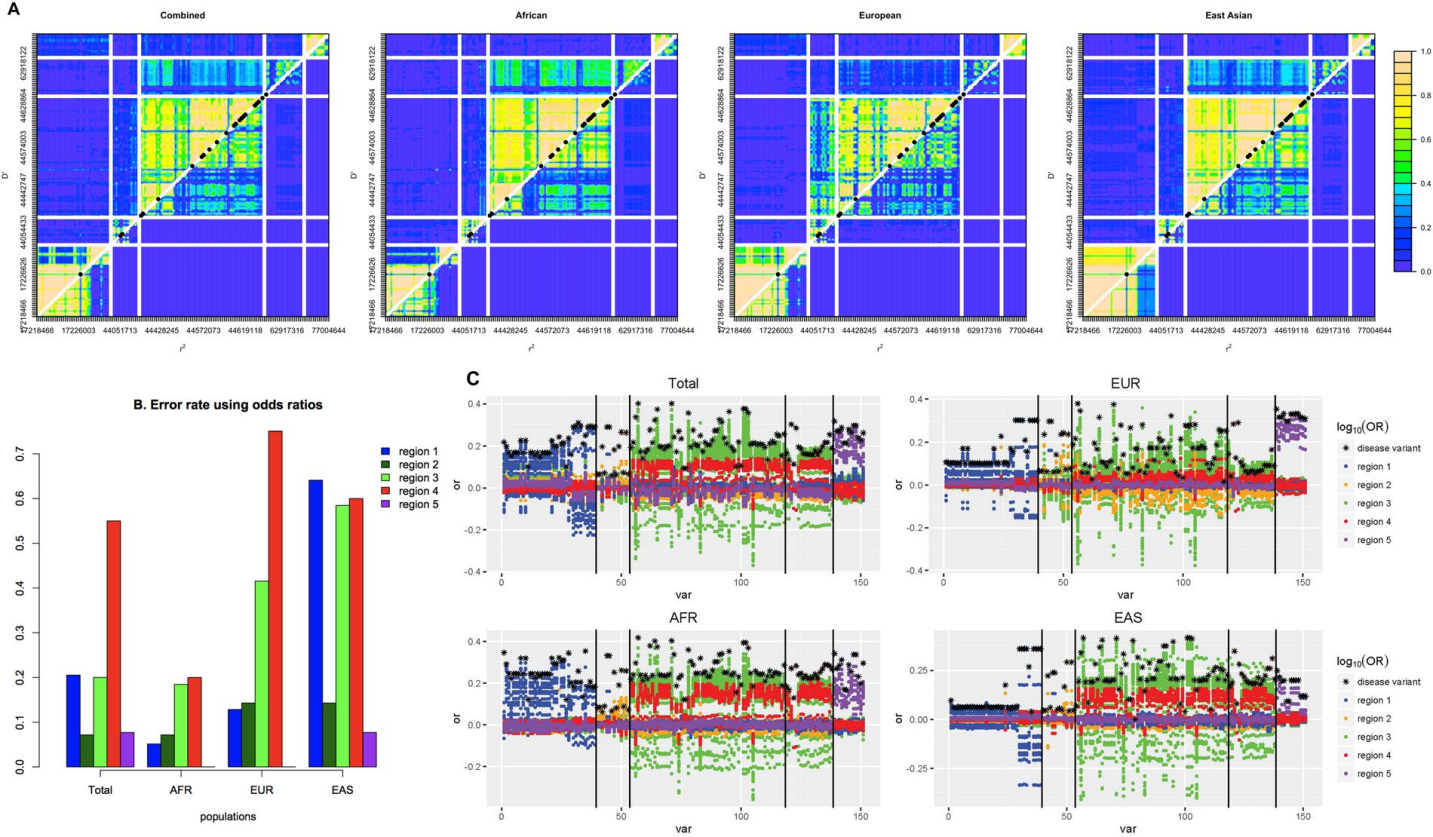
Coincident LRLDs in chromosome 17: (**A**) Linkage disequilibrium; (**B**) Error rates using odds ratios; (**C**) Plots of log of odds ratios depending on regions for the total, AFR, EUR, and EAS populations.

Four LRLD regions and a fifth region without LRLD of chromosome 17 were selected to examine the genome-wide effect of LRLDs on association studies. Population specificity was also observed in the LD plot. Extensive LD was observed in EUR between regions 2 and 3, which are close to each other, and the strongest extensive LRLDs were observed between regions 3 and 4 in AFR. The LD pattern in each region differed among the populations. The LRLD variants were in strong LD within each region. In a combined population, artificial LRLDs were observed due to population substructure (Fig. 4A), as some of the variants showed large differences in allele frequencies between populations. The fifth region without any significant LRLD showed LRLD in the combined population with several variants in regions 1, 3, and 4. When examining the combined population, most variants throughout region 1 were in LRLD with a variant in region 3, with a few LRLDs in this region exclusively observed in AFR. Those variants lead the LRLDs in the fifth region exclusively in the combined population, with large differences in the allele frequencies of the variant in AFR (0.19) vs EUR (0.65) and EAS (0.87). In region 4, no variants were present that had a minor allele frequency greater than 0.1 in either the EUR or EAS population within 30,000 bp before the LRLD variant rs11650755. The variant is located in the 5′UTR of *LRRC37A3* (leucine rich repeat containing 37 member A3), which is mostly expressed in the testis^27^. More studies are necessary to reveal the reason for the genomic desert of common variants in this region for EUR and EAS populations.

Figure 4B indicates the proportion of correct detections of disease variants using odds ratios, indicating that the disease variant shows the highest odds ratio. Again, region 5 contained no LRLD variants and showed consistently low error rates in detecting disease variants using odds ratios. The results differed among the populations. Overall, AFR exhibited the lowest error rates, and EAS presented the highest error rates. Region 2 in LRLD with region 1 showed lower error rates than other LRLD regions. The variants of other regions in LRLD showed clear inflation of odds ratios in their corresponding LRLD regions. In particular, the region 4 presented consistently inaccurate results due to the variants of region 3 in LRLD. As shown in Fig. 4C, the region showed consistently high odds ratios of variants in region 3, resulting in the false identification of disease variants using odds ratios. However, regarding the other regions, the errors primarily arose from nearby variants in LD rather than from far variants in LRLD.

**Figure 5:**
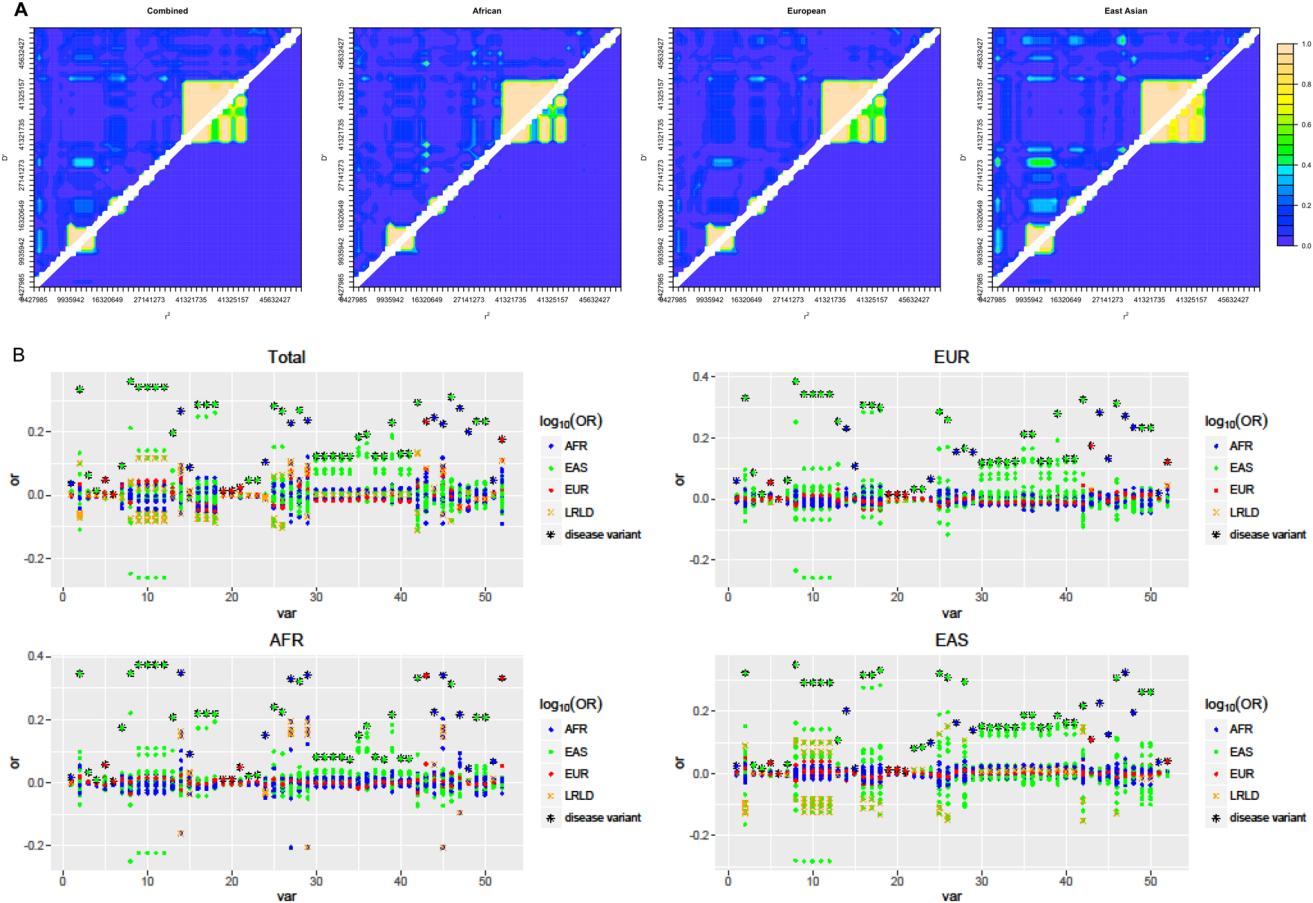
Chromosome-wide impact of LRLDs in chromosome 21: (**A**) Linkage disequilibrium; (**B**) Plots of log of odds ratios of each LRLD variants for the total, AFR, EUR and EAS populations.

In total, 52 LRLD variants from chromosome 21 excluding centromeric regions were selected to examine their genome-wide impacts on association studies. Among them, no LRLD was found concurrently in two or three populations for chromosome 21. Eleven LRLD variants were detected in AFR, seven LRLD variants were detected in EUR, and 34 LRLD variants were detected in EAS. Both strong LRLD and LD between close regions were observed (Fig. 5A). Similarities among LD plots in close regions were also observed among populations; however, LRLD differed among the populations, although there were similar trends between EUR and EAS.

The seven LRLD variants in EUR had low allele frequencies in all three populations. When the LRLD variants are disease variants, these variants typically exhibited the highest odds ratios, which is the case for the 11 LRLD variants in AFR. Among the seven LRLD variants in EUR, one variant showed several other variants having higher odds ratios than the actual disease variants in both EUR and EAS. Among the 34 LRLD variants specific to EAS, six had other variants with higher odds ratios than disease variants among various populations including the total population. All the LRLD variants with wrong identification of disease variants were located in strong LD region among nearby variants. The incorrect identification of disease variants varied among the populations. As shown in Fig. 5B, the variants that are not disease variants but were in LRLD with the disease variants showed more changes in their odds ratios than other variants not in LRLD with the disease variants. However, the increments or decrements were not usually as large as the variants in local LD with the disease variants, indicating that local LD can cause severer problems in detecting actual disease variants. Even when disease variants were detected, the fact that disease variants in strong LRLD caused false signals of variants at a long distance remains a serious problem.

To examine the actual impacts on GWAS, the significant variants from GWAS catalogs (version 1.0, July 17, 2018) were examined for concordance with LRLD variants. For 88 LRLDs, one of the LRLD pairs were matched with the GWAS variants. For none of the LRLDs showed were both LRLD variants matched with the GWAS variants. However, among 72,633 GWAS variants, only five were among the 88 LRLDs, indicating that the variants are LRLD hotspots. Three variants were located in chromosome 6 (32,658,715, 32,663,606, and 32,680,379), on *HLA-DQA1*, *HLA-DQB1* and *MTCO3P1*. This HLA area is strong LRLD hotspots in EUR and EAS with the region, 5,000,000 apart from the HLA area. The remaining two variants were in chromosome 11 (205,198 and 229,977) located on *BET1L* and *STRT3*, and were in LRLD with the region after the centromere. The most recent version of GWAS catalog (version 1.0.2) provides a slightly increased numbers in concordance (88 LRLDs to 102 LRLDs and 5 LRLD variants to 13 LRLD variants); however, all the variants located on the same regions of 6p21.32 and 11p15.5.

GWAS variants are selected for capturing the majority of regional variants based on LD^33^. Therefore, because these variants might represent a marker signal rather than the actual disease variant^34^, only five variants exactly matching variants with LRLDs might be observed. As shown in Supplementary Fig. 3, most significant GWAS sites were plotted alongside LRLD regions, indicating that GWAS sites might be influenced by LRLDs. Interestingly, all the LRLDs harboring GWAS variants were significant only in EAS; however, only one of them (chromosome 11, 205,198, 3′UTR in *BET1L*) involved GWAS with individuals of East Asian ancestry. Most of the GWAS variants were based on individuals of European ancestry, and one on individuals who were Hispanic/Latino. Those LRLD regions were significant in both EUR and EAS. Therefore, although the LRLDs were significant only in EAS, the LRLD regions might influence EUR similarly.

## Discussion

The current study revealed clear population differences in LRLDs, indicating the existence of population-specific long-range interactions. AFR showed fewer LRLDs yet spread widely throughout chromosomes, whereas EUR and EAS showed more LRLDs focused on specific regions. Severe bottlenecks in the past population histories of EUR and EAS compared with those of AFR might explain the concentrated LRLDs in EUR and EAS in certain regions^9,35^. However, unlike LD between close regions, LRLD can easily be disrupted unless there are specific forces acting to maintain LRLD. The main events of recent human populations are rapid population expansions rather than bottlenecks^36^, which are much more severe in EUR and EAS than in AFR^24^. Given that recent rapid population expansions would result in reduced LD^3^ and bottlenecks should influence the whole genome rather than specific regions, the observation of strong, irregular, focused LRLD in EUR and EAS might indicate slight interacting forces, considering the properties of LRLD and the more recent changes in human population sizes.

For the correction of multiple testing, FDR corrections were applied to identify the true LRLDs. FDR corrections only indicate the number of true LRLDs rather than the actual LRLDs. If 10 LRLDs are significant after FDR correction, it indicates that there are 10 true LRLDs. True LRLDs are likely the most significant LRLDs; however, due to sampling error, true LRLDs might remain in the group of FDR-corrected p-values > 0.05. Therefore, when studying true long-range interactions, the target long-range interactions must be assessed as LRLDs with and without FDR corrections. To reduce the possibility of investigating incorrect LRLDs, large sample sizes and consideration of the actual functionality of LRLD regions would be helpful. Due to population specificity, comparisons between populations might not be helpful for identifying true LRLDs.

The current study replicated two regions previously reported to have three extended LD blocks in European populations^18^: a 25.5~33.5 Mb region on chromosome 6 (LRLDs in EUR and EAS) and the region near the centromere of chromosome 11 (LRLDs in AFR and EUR). Interestingly, the region of 8–12 Mb on chromosome 8 harboring an inversion polymorphism was not the region of LRLD in the current study. The strong LRLDs surrounding centromeres indicate the accuracy of the current results; however, the possibility that gene interactions occur between genes before and after centromeres, especially in chromosomes 9 and 11, cannot be excluded. The LRLD patterns of AFR were the most diverse, and the LDLR patterns of EUR and EAS showed LRLD hotspot regions. The results support the possibility that weak long-range hitchhiking may also occur in the human genome, which was observed in experimental *Drosophila melanogaster* populations^37^.

Although at least parts of LRLDs in relatively close distance are caused by long-range hitchhiking, such strong directional selection pressure cannot explain the majority of chromosome-wide LRLDs that were seen in the current study. Structural variants can be an alternative explanation; however, considering their rarity in terms of their allele frequencies and their limited lengths as shown in the Results, their impacts cannot fully explain the chromosome-wide LRLDs. One plausible explanation could be that two distant strong directional selection pressures make LRLDs in a chromosome. In this case, it is usually difficult to have functional relations between LRLD variants, which is not the case in the current study. In particular, as shown in Fig. 4A, the LRLD variants in the regions 1, 2, and 4 showed weaker LD within the LD block, indicating that the LRLD variant is not the leading variant of directional selection. A more plausible explanation could be a weak interaction between LRLD variants in related genes as indicated previously. Evidence of weak interactions between coding and regulatory variation was provided in a previous study^38^. They observed purifying selection of gain-of-expression quantitative trait loci (eQTLs) and stronger LD between eQTLs and nonsynonymous variants. Although their observation mainly focused on close cis-regulation within a gene, the phenomena can be widened in a chromosomal level between a distant trans regulatory region and a coding gene. However, the question still remains as to how the weak interaction survives from extensive recombination events.

The derived allele frequencies of LRLD variants in coding sequences as well as the remaining genic regions (5′UTR, 3′UTR, intron, and noncoding gene) did not show any particular properties in their distributions such as the changes in double-heterozygote fitness^38^. Considering the functional similarity and long distance between genes in LRLDs, the weak interaction is neither solid nor stable. Whenever the interacting force disrupted by environment or other strong interfering force exist, the interaction may vanish, which can be the reason why there are a few population concurrent LRLDs. Further studies would be necessary for a detailed mechanistic explanation on LRLD formation. Interestingly, many LRLD variants were LRLD hotspots. Perhaps, a number of those variants may have pleiotropic effects in their phenotype expression as shown recently^39^.

Migrations can induce population substructure that produces LRLD artifacts^2,11^. As shown in Fig. 1, population substructure due to migrations can provide the LRLD artifacts if the allele frequency differences are large enough. Although differences in the allele frequencies within a continent are small, the estimation of subpopulation structure can still detect subpopulation structures within each population based on setting the number of expected subpopulations^9,40^. In EUR and EAS, if the cluster is equal or less than six, almost homogeneous population without obvious subpopulation can be seen; however, AFR showed more various structure even with a small number of clusters^9^. As long as huge differences in allele frequencies between subpopulations are not observed, the LRLD artifacts from subpopulation structure may influence partially at most. Another serious concern may arise when the current continental population references from the 1000 Genomes Project may represent their genomic variation imperfectly, especially again for Africans. A recent study on Southern African population revealed the concern although their study is based on a cohort of HIV positive children^14^. With an enlarged study of populations of human genome, analyzing each subpopulation within a continent may exclude those concerns, providing more elaborate results on each population.

The present study demonstrated that LRLDs can actually influence the results of association studies; however, in the present study, the impacts were not extensive, presenting limited influence most of the time. This limited influence occasionally leaves false signals in the counterpart region of LRLDs. Because previous GWASs did not evaluate whether a signal in a region arose from LRLD, both true and false signals would have been detected. Interestingly, as shown in Supplementary Fig. 3, the significant sites from GWAS catalogs (version 1.0) mostly overlapped with LRLD sites, although the GWAS hits were distributed in a genome-wide manner^41^. More serious problems might arise when examining genome-wide gene interactions^15^. Although false signals through LRLD might not be frequent, their potential existence must be considered in GWASs. Based on the disease model, if the disease allele frequency is high, the increments or decrements of the disease genotype frequencies are small, resulting in low odds ratios^42^. As shown in Fig. 4C, the small odds ratios of EAS resulted from the large disease allele frequencies, which also increased the error rates when testing single variant associations, which have served as the typical basic method in most genome-wide associations.

The functional relevance of structural variants has been revealed^43–45^. Although the number of LRLDs with structural variants is not large and their lengths were not sufficiently large to influence the LRLDs, the possibility that rare functional structural variants in strong LD with nearby variants could interact with a distant variant^46^, influencing the LRLDs detected in the current study, still cannot be excluded. As shown in the Results, although their lengths and allele frequencies were limited to create the chromosome-wide LRLDs, 44.1% of LRLD variants were located on structural deletion or inversions. These structural variants can cause false signals not only in variants on and near themselves but also in distant variants in LRLD with them. In this case, comprehensive analyses of all variants including structural variants must be conducted to correctly identify disease variants. Because LRLDs with structural variants also showed substantial population specificity and most of them were rare variants, the population specificity should also be considered in the analyses.

In the present study, when testing LD, physical distance was used instead of genetic distance. Because recombination rates differ depending on the chromosomal region^47^, an adjustment using genetic distance might produce more accurate results. However, although a physical distance of 5 × 10^6^ bp was used, most of the LRLDs showed far greater distances between the variants chromosome-wide rather than the distance close to the minimum, as shown in Fig. 2. Therefore, most of the LRLDs in the current study would remain LRLDs even when using the genetic distance in each chromosome. The results of the current study rely on the phasing of the 1000 Genomes Project data^9^. Given that the project constructed high-quality haplotypes through a staged approach^48^, the accuracy of the LRLD detections can be considered high. To ensure accuracy, composite genotypic disequilibria can be applied instead^17^; however, without excesses in two-double heterozygotes, the differences in the results would be minimal. The gene interactions in the current study are based on allelic associations. Genotypic association approaches could be further applied^16,17,49^. More importantly, further functional validations are necessary to identify actual long-range interactions.

Although a substantial number of samples have been genotyped in numerous GWASs, it turned out that the most widely used Affymetrix 6.0 SNP chip does not include any of the LRLD positions revealed in the current study. The Illumina HumanExome chip did include a substantial number of overlapping variants in LRLD. Based on the control data from the WTCCC2 (Wellcome Trust Case Control Consortium 2), there were 659 pairs of matched LRLD positions; however, the available LRLD positions were not meaningful because most of the pairs (654) were located surrounding centromeres in dense LRLD regions. The remaining four LRLD regions, were located within dense LRLD regions on chromosome 6 specific to EUR and EAS, and one LRLD was located near the centromere of chromosome 10, a dense LRLD region that is common among all populations (Fig. 2). Therefore, the direct impacts of LRLDs would not be observed in most GWASs; however, the indirect impacts of LRLDs would influence the nearby variants of the LRLD variants (Fig. 4B). For further examinations of direct LRLD impacts on actual GWASs, two different sequencing data of similar populations would be helpful.

For convenience, the current study assumed one disease variant in a chromosome, which is typically not true. By considering the complex genome-wide influences of multiple regions in association studies, true signals can be identified regardless of LRLD. Interestingly, considerable population specificity of LRLD was observed at the chromosome-wide level with substantially reduced LRLDs than the expected numbers occurring concurrently in two or three populations when the centromeric LRLDs were removed. The results indicate the possibility of population-specific, long-range interactions in the human genome, which is also supported by the observation of increased gene regions in LRLD, excluding possible false positives in the recombination cold region of centromeres.

## Methods

### Long-range linkage disequilibrium in the human genome

The phase 3 data of the 1000 Genomes Project were used in the current study (http://ftp.1000genomes.ebi.ac.uk/vol1/ftp/release/20130502/)^9,10^. Unrelated individuals from three populations were selected: 504 Africans (AFR), 504 East Asians (EAS), and 503 Europeans (EUR). To avoid population substructure or population admixture among the AFR individuals, only Africans residing in Africa were included; i.e., individuals of African ancestry in the Southwest US (ASW) and the African Caribbeans in Barbados (ACB) were excluded. Therefore, AFR includes Yoruba in Ibadan (YRI); Luhya in Webuye, Kenya (LWK); Gambian in Western Divisions in the Gambia (GWD); Mende in Sierra Leone (MSL); and Esan in Nigeria (ESN). The current study focused on variants in 22 autosomal chromosomes with allele frequencies between 0.1 and 0.9 in each of the three populations. The cutoff was set conservatively to exclude the possibility of observing LRLD due to random sampling. For multi-allelic variants, the allele with the largest minor allele frequency among all populations (a total of 2,504 individuals) was selected for analysis.

To examine LRLD, the distance between variants was defined as 5 × 10^6^ bp, considering the sizes of small chromosomes. This distance is substantially increased compared with the distance of 2.5 × 10^4^ bp considered in a previous study^7^. The final numbers of estimated variants and LRLD by gene region are summarized in Supplementary Table 1. Similar to a previous study^50^, the genic and nongenic regions were coded as follows: (1) CDS; (2) 5′ untranslated region (UTR); (3) 3′UTR; (4) noncoding gene region; (5) intron; (6) the region between −1,000 bp and the gene start site; (7) the region between +1,000 bp and the gene end site; (8) the region between −5,000 bp and the gene start site; (9) the region between +5,000 bp and the gene end site; and (10) the remainder of the nongenic regions based on ENSEMBL Perl API^32^. The distance provides recombination rates of 0.0543 based on the Haldane map function and 0.0572 based on the Kosambi map function assuming approximately 1.15 cM for 1 Mb^51^. The structural variants were detected when named starting “esv” in the 1000 Genomes Project data using a perl script. The calculation of proportions were conducted using basic functions of the R Project for Statistical Computing (https://www.R-project.org/).

Because the chi-square test was slightly less affected by sampling than was Fisher’s exact test (Fig. 1) and because it is more computationally favorable, the chi-square test was used for detecting LRLD. Since the sample sizes of the study populations (504 and 503) are higher than the sample size of the 500 considered in Fig. 1, slightly reduced false-positive or false-negative LRLD detection would be expected. LRLD was considered to be detected only when the corrected p-values had a FDR less than 0.05, unless indicated otherwise. The FDR calculation was performed based on the basic function of the R Project for Statistical Computing. All the processing of vcf format data and calculations were performed using a C++ code based on the basic distributional relationships^52^ with MPI parallelization. Detected LRLDs were plotted using the R package “circlize”^53^. Gene enrichment analysis was conducted based on the protein annotation through evolutionary relationship (PANTHER) classification system, which provides the web-based interface^28,29^.

### Odds ratio calculations based on a dominant model

To examine the odds ratio changes due to LRLD, the genotype data for the regions of LRLD were extracted from the 1000 Genomes Project data for the AFR, EUR and EAS populations described above. In most cases, if one LRLD variant was detected in a region, the variants in the region within ±10,000 bp of the target variant were examined. By adopting a sufficient causal component model for complex diseases^54^, a complex disease with two factors of environment (E) and gene-environment interaction (G_E_ × E_G_) was modeled. In this model, the population environmental factor (E) was 0.03, indicating that an average of 30 among 1,000 individuals had the factor and were affected by the factor. In addition, the population environmental factor interacting with genes (E_G_) was 0.05, indicating that an average of 50 individuals had the factor and would be affected if they had the gene factor interacting with E_G_ (G_E_). At a gene frequency of 1 for the gene interacting with the environment (G_E_), the population lifetime incidence (E + G_E_ × E_G_) would be 0.08. The proportion of gene-environment interaction was calculated for each disease variant for each population, and the derived genotype frequencies in the case population were calculated as described elsewhere^42^. Because dominant and recessive models showed similar results in a previous study^42^, a dominant model for one interacting gene was assumed. The frequency of disease alleles varied by variants and populations. The computations were based on the R Project for Statistical Computing (https://www.R-project.org/).

### The impacts of LRLD regions on case-control associations

To examine the impact of LRLD on case-control associations, chromosomes 17 and 22 were selected, which were the smallest and with the evenly distributed population-specific LRLDs. Four regions in chromosome 17 that showed coincident LRLD in all three populations and an additional region that was irrelevant to LRLD (76,995,000-77,005,000) were selected to examine the genome-wide effect of LRLD in association studies. For each region, the region ±10,000 bp from the LRLD variants common among all three populations was considered. However, region 4 was ±30,000 bp from the LRLD variant, given that there were only five variants within the region ±20,000 bp. For examining the genome-wide impacts of LRLDs, chromosome 21 was selected due to its small size and proper population diversity, in which each population has at least a few LRLDs. Chromosome 21 showed a high number of LRLD surrounding the centromere; therefore, the LRLDs surrounding the centromere were excluded from the analyses, resulting in 52 variants.

## Supplementary information

Supplementary Information

Supplementary Dataset 1

Supplementary Dataset 2

## Data Availability

All the data generated during this study were included in this article and the Supplementary Information. Further information can be available upon request.
