# Supplementary material for: Population-specific long-range linkage disequilibrium in the human genome and its influence on identifying common disease variants
Source: Sci Rep. 2019 Aug 6;9:11380. doi: 10.1038/s41598-019-47832-y (PMC6684625; doi:10.1038/s41598-019-47832-y)
Supplement: Supplementary file 1 — Supplementary Information [file 41598_2019_47832_MOESM1_ESM.pdf]

Supplementary Information

Population-specific long-range linkage disequilibrium in the human genome and its influence on identifying common disease variants

Leeyoung Park*

***Natural Science Research Institute, Yonsei University, Seoul, Korea 120-749;**

Corresponding author: Leeyoung Park

Address: Natural Science Research Institute, Yonsei University, 134 Shinchon-Dong, Seodaemun-Ku, Seoul, Korea 120-749

Phone: (82)2-2123-3530

Fax: (82)2-313-8892

Email: lypark@yonsei.ac.kr

Supplementary Figures: 3

Supplementary Tables: 2

Supplementary Datasets (separate files): 2

Supplementary Text

Supplementary Figure 1. Chromosome-wide coincident LRLD of AFR-EUR, AFR-EAS, EUR-EAS, and in all three populations.

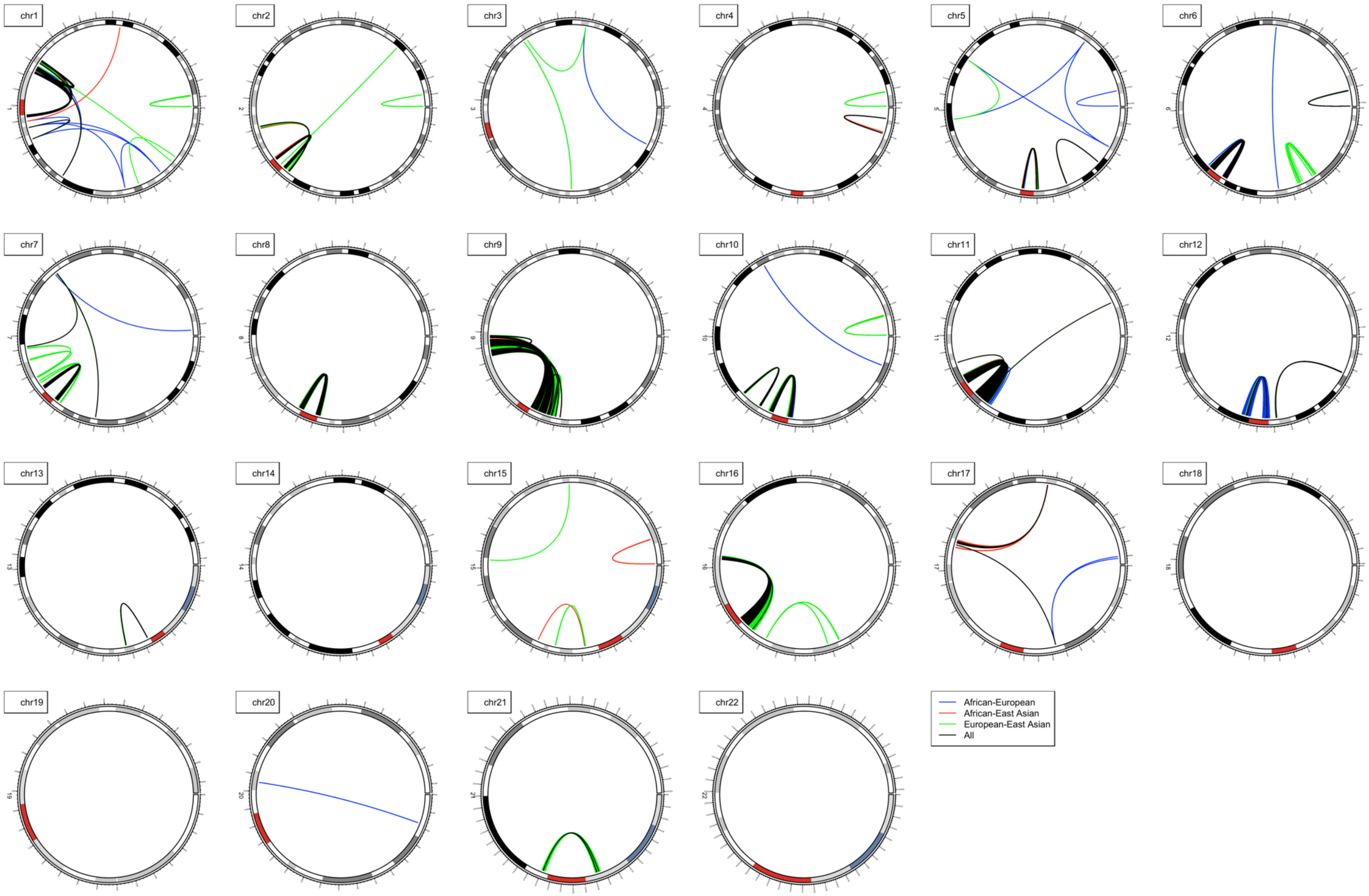

Supplementary Figure 2. Linkage disequilibrium of CDS-CDS LRLD regions in chromosome 9 (red dotted lines indicate the LRLD variants).

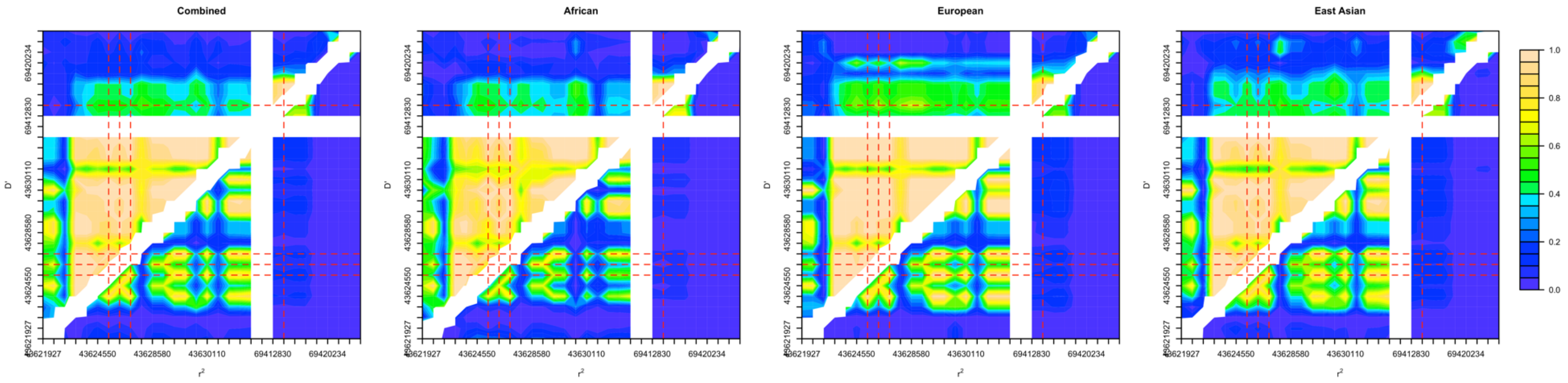

Supplementary Figure 3. The combined plots with GWASs and LRLDs (the red bar indicates the centromere, and the black dots indicate the positions and log-scale significances of GWAS variants).

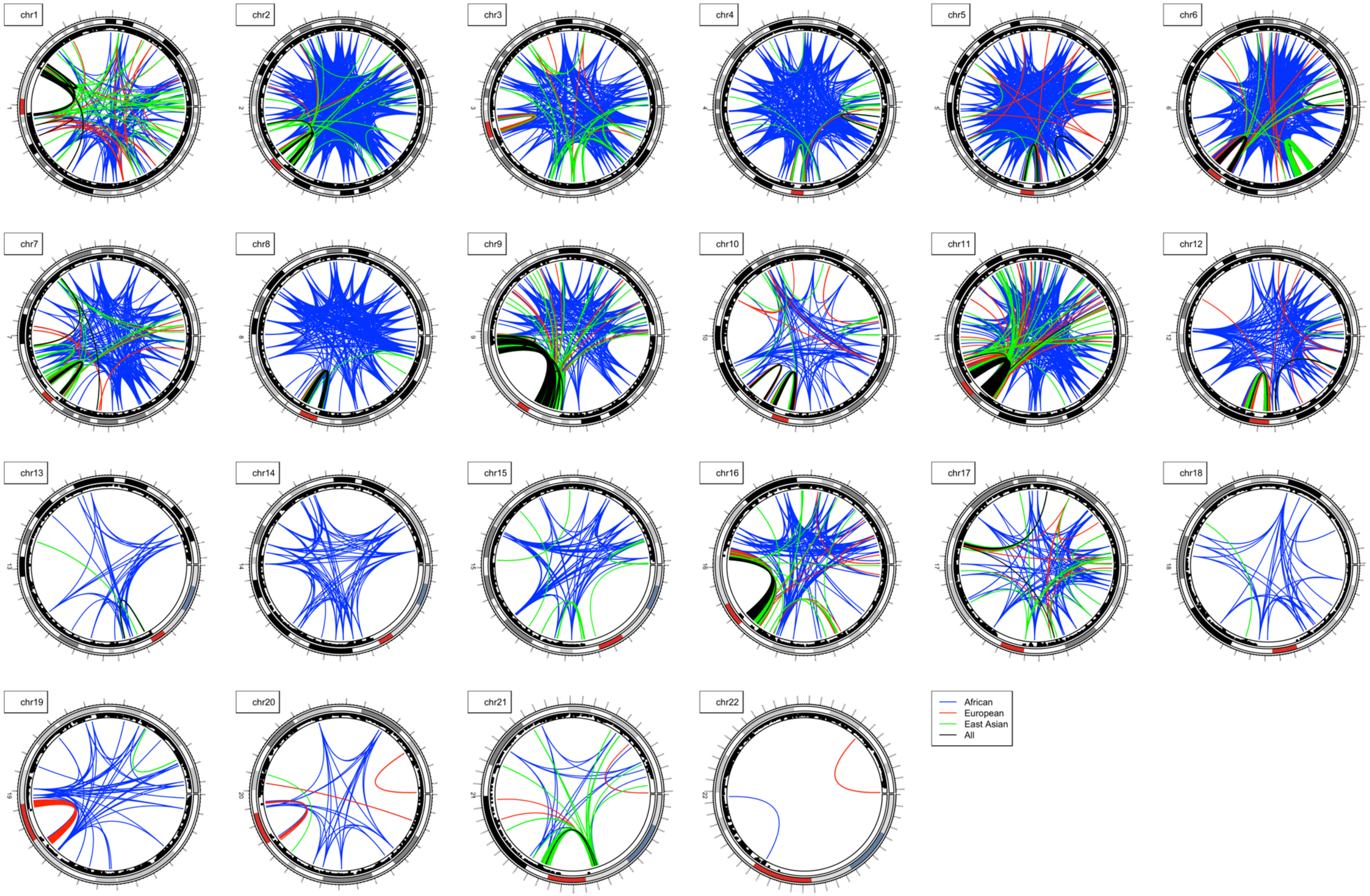
chr1
chr2
chr3
chr4
chr5
chr6
chr7
chr8
chr9
chr10
chr11
chr12
chr13
chr14
chr15
chr16
chr17
chr18
chr19
chr20
chr21
chr22
African
European
East Asian
All

Supplementary Table 1. Summary of total examined variants based on gene regions and total LD calculations.

chr	1	2	3	4	5	6	7	8	9	10	Total variants	LD calculations
1	1738	694	3986	4101	114560	3804	4206	11021	10217	106933	261260	32608746160
2	1177	529	2542	3511	119918	2582	2730	8631	7584	128466	277670	36918179799
3	967	546	2998	2936	112141	1880	2068	6358	5912	105134	240940	27545318850
4	730	408	1871	2730	108797	2057	2265	7138	6951	121561	254508	30674615611
5	800	396	1867	2569	87171	2205	2221	7114	6302	107139	217784	22400700893
6	1486	560	3560	3734	86415	3304	3567	10301	8260	119050	240237	26930721524
7	789	380	2049	2846	79188	2159	2534	7445	6936	101356	205682	19800406061
8	566	328	2335	2211	85391	1548	1737	5392	5270	79471	184249	15740311905
9	646	276	1315	1923	61181	1671	1750	5748	4780	67280	146570	9826597065
10	661	258	1340	2050	75605	1801	1870	5798	5247	78725	173355	13908084676
11	1129	799	1876	3015	69369	2589	2694	8709	6805	77668	174653	14123466587
12	864	1202	2647	3074	79462	1869	1936	5782	5549	57818	160203	11869446663
13	276	124	808	1475	43128	1129	1165	3658	3653	73028	128444	7407998956
14	725	392	2162	1748	51806	2079	1761	4699	4368	42862	112602	5645176831
15	597	380	1470	2015	54479	1034	1139	3206	2839	31389	98548	4264872609
16	708	380	1522	2656	55023	1281	1394	4045	3250	36410	106669	4911914423
17	970	512	1581	2621	46987	1612	1581	4584	3303	30673	94424	3906368813
18	299	235	989	1369	38056	967	1004	3152	2788	48353	97212	4128314943
19	1648	864	2416	2878	45508	2331	2326	6139	4686	17953	86749	3145956563
20	387	149	622	1096	26317	1125	1374	3703	3585	37671	76029	2430174776
21	184	69	339	834	12315	791	842	2361	2144	33179	53058	1040634530
22	355	171	673	1897	18010	1175	1205	3159	2461	17831	46937	808006083
sum	17702	9652	40968	53289	1470827	40993	43369	128143	112890	1519950	3437783	300036014321

Supplementary Table 2. Proportion of variants depending on gene regions:

A. Proportions of total, LRLD, and LRLD-hotspot variants;

	1	2	3	4	5	6	7	8	9	10
Total	0.00512	0.00279	0.01191	0.01523	0.42852	0.01179	0.01252	0.03714	0.03274	0.44223
LRLD	0.00529	0.00200	0.00487	0.02119	0.40921	0	0	0	0	0.55743
LRLD hotspots	0.00657	0.00213	0.00478	0.01905	0.39823	0	0	0	0	0.56924
wo/surrounding centromere	0.00742	0.00352	0.00555	0.02888	0.47518	0	0	0	0	0.47945
wo/centromeric region	0.00851	0.00567	0.00945	0.03257	0.50467	0	0	0	0	0.43912
variants with extreme frequencies in AFR subpopulation	0.00737	0.01474	0.03317	0.01843	0.48649	0	0	0	0	0.43980

B. Proportions of LRLD variants of each population;

	1	2	3	4	5	10
Total	0.01173	0.00059	0.00201	0.02008	0.41834	0.54725
AFR	0.01292	0.00030	0.00103	0.02053	0.42508	0.54014
EUR	0.01126	0.00066	0.00180	0.01991	0.42225	0.54413
EAS	0.01228	0.00034	0.00136	0.02140	0.43745	0.52717

C. Proportions of LRLD excluding surrounding centromeres;

	1	2	3	4	5	10
Total	0.00224	0.00093	0.00090	0.02461	0.30935	0.66197
AFR	0.00227	0.00076	0.00062	0.02481	0.31575	0.65580
EUR	0.00150	0.00108	0.00075	0.02582	0.32690	0.64395
EAS	0.00227	0.00070	0.00070	0.02246	0.31559	0.65828

D. Proportions of LRLD excluding on and near centromeres.

	1	2	3	4	5	10
Total	0.00548	0.00539	0.00558	0.04246	0.45475	0.48635
AFR	0.00586	0.01518	0.00886	0.01856	0.46631	0.48524
EUR	0.00821	0.00792	0.00751	0.06640	0.49671	0.41325
EAS	0.00257	0.00304	0.00294	0.02155	0.39585	0.57405

Supplementary Dataset 1 (SupplTableesv_unique_info_alt.xlsx). LRLD structural variants: A. position, length, variant information, allele frequencies, and gene information; B. Summary of population specificity.

Supplementary Dataset 2 (SupplTable4.xlsx). Summary of CDS-CDS LRLDs.

Supplementary Text

Detailed description regarding Figure 3 involving NMD transcript variants

In CDS variants, the proportions of functional variants including any frame shift, missense, inframe deletion, stop codon, splice, and NMD transcript variants were examined based on ENSEMBL Perl API. The proportion of functional variants among the total calculated CDS variants increased from 0.476 without NMD variants and 0.618 with NMD variants in total calculated variants to 0.650 and 0.707 in the LRLD variants, respectively. Almost same proportions were observed when considering only missense and frameshift variants. When excluding LRLDs surrounding the centromere, the proportion of functional variants among the total calculated CDS variants increased slightly to 0.662 without NMD variants and to 0.725 with NMD variants, respectively. When excluding LRLDs on and near the centromere, the proportion decreased to 0.593 without NMD variants and to 0.691 with NMD variants. Similarly, 97% and 98% of functional variants with and without NMD variants, respectively, were observed more than once in LRLDs excluding those surrounding the centromere; excluding LRLDs on and near the centromeres, the corresponding percentages were 95% and 96%. When considering only missense and frameshift variants, 100% of the functional variants were observed more than once in LRLDs excluding LRLDs either surrounding or on/near the centromere.

As shown in Figs. 3A and 3B, the proportion of variants located in the 5' UTR was the lowest for all LRLD variants and increased when LRLD variants on and near the centromere were excluded, yielding a higher proportion than that among the total estimated variants. Similar trends were observed in the proportions of variants in noncoding regions and in introns. The proportion of variants located in the 3' UTR showed similar trends as that in the 5' UTR; however, the proportion of variants in the 3' UTR was the highest among the total

estimated variants. Interestingly, there was no LRLD variant detected in the ±5000-bp gene regions (coded as 6, 7, 8, and 9). It is surprising that even smaller proportions of gene regions were observed as LRLD variants. The proportions of variants in nongenic regions showed opposite trends to the proportions in any gene regions.

The total number of calculations to detect LRLD was 300,036,014,321, and LRLD detection was considered if LRLD was observed in at least one population. The proportion of variants in genic regions coded 1 through 5 was 0.4587 (Supplementary Material, Table S1). The proportion in any gene regions slightly decreased to 0.4431 for LRLD variants, which resulted in the expected number of LRLD with both LD positions in genic regions of 3,124,330. The actual observed number was 3,335,296, which is slightly higher than the expectation. The proportion in any gene regions increased to 0.5215 and 0.5623 for LRLDs excluding those surrounding centromeres and excluding those on and near centromeres, respectively. For LRLDs with both LD positions in genic regions, the observed number of LRLDs excluding variants on and near the centromere was 38,817, which is again slightly higher than the expected value of 38,145; however, the observed number of LRLDs excluding those surrounding the centromere was 151,009, which is less than half the expected number of 330,708. The result indicates that there are many more LRLDs between a genic region and a non-genic region for LRLDs excluding those surrounding centromeres, most of which were from chromosome 9 (81.4% of genic-nongenic LRLDs), as shown in Fig. 2. After excluding all of the regions on and near the centromere, the same trend of slightly higher observed genic-genic LRLDs than expected was found, confirming that most of the genic-nongenic LRLDs in chromosome 9 were removed.

There was a slight increase in the proportions of CDS and 5’UTR in LRLD hotspot variants that were found to be involved in LRLD at least 100 times, as presented in Fig. 3A; however, other gene regions showed slightly decreased proportions with an increasing proportion

of non-genic regions (Supplementary Table 2). The gene proportions including repeated variants in LRLD were different from those of unique variants in LRLD. As shown in Supplementary Material, Table S3B, the proportions of CDS variants was almost twice as large as the proportion of CDS unique variants in all of the populations, indicating that CDS variants were repeatedly involved in several LRLDs. The results are consistent with the findings that 92% of the functional CDS variants of frame-shift and/or missense variants were involved in LRLD more than once and were predominantly observed as LRLD hotspots. For the proportions excluding centromeric regions, 100% of the functional CDS variants were involved in LRLD more than once. These results indicate the possibility of functional LRLD due to long-range gene interactions.

As shown in Fig. 3B and Supplementary Material, Table S3, the results differed slightly among populations. The EUR population showed a slightly smaller proportion of CDS variants and much larger proportions of 5'UTR and 3'UTR variants than the other populations. However, when excluding LRLDs having variants on and near the centromere, the proportion of CDS variants increased in the EUR population (Supplementary Material, Table S3D). The proportions of other regions also showed slight yet clear population differences as shown in Supplementary Material, Table S3. These population differences indicate the possibility of population-specific long-range interactions.
